# Energy Drinks and Adverse Health Events in Children and Adolescents: A Literature Review

**DOI:** 10.3390/nu15112537

**Published:** 2023-05-29

**Authors:** Pengzhu Li, Nikolaus Alexander Haas, Robert Dalla-Pozza, André Jakob, Felix Sebastian Oberhoffer, Guido Mandilaras

**Affiliations:** Division of Pediatric Cardiology and Intensive Care, University Hospital, LMU Munich, 81377 Munich, Germany; pengzhu.li.extern@med.uni-muenchen.de (P.L.); nikolaus.haas@med.uni-muenchen.de (N.A.H.); robert.dallapozza@med.uni-muenchen.de (R.D.-P.); andre.jakob@med.uni-muenchen.de (A.J.); felix.oberhoffer@med.uni-muenchen.de (F.S.O.)

**Keywords:** energy drinks, caffeine, cardiovascular, neuropsychiatric, children, adolescents, adverse health events

## Abstract

This review aims to investigate and summarize adverse health events in children and adolescents associated with energy drink (ED) consumption, while also exploring the impact of simultaneous trigger factors and/or preexisting health conditions. We searched the database of PubMed, Cochrane library, and Web of Science for cases associated with ED consumption in minors up to 9 May 2023. The literature written in English met inclusion criteria if patients were <18 years of age and the ED consumption was confirmed. Records, relevant articles, and reports that met all inclusion criteria were fully read by two researchers. In total, 18 cases reporting adverse health events were included. Of those, 45% affected the cardiovascular system, 33% the neuropsychological system, and 22% other organ systems. In 33% of cases, additional triggers were reported. In 44% preexisting health conditions were present. This literature review suggests that ED intake may well be associated with adverse health events in minors. The cardiovascular and the neuropsychiatric systems seem to be predisposed. ED consumption in combination with potential trigger factors or in the presence of preexisting health conditions appears to be critical. To prevent adverse health events in the future, children and adolescents should be informed about risk factors and responsible consumption behaviors.

## 1. Introduction

Energy drinks (ED) are sweetened beverages containing stimulant compounds, such as caffeine or guarana [1] and are marketed as mental and physical enhancers [2]. Despite their well-known side effects on the cardiovascular system (e.g., arterial hypertension and cardiac arrhythmias) [3], ED consumption behavior remains very high, especially among teenagers [4]. Data from the NOMISMA-ARETÉ Consortium for the European Food Safety Authority (EFSA) demonstrated that the highest prevalence of ED consumption is reported in adolescents (68%), followed by adults (30%) and children (18%) [5]. Of those underaged ED consumers, the prevalence of high chronic consumption was estimated at 12% for adolescents and 16% for children, and the prevalence of high acute consumption at 12%, respectively [5].

In line with their increasing popularity among minors, the literature shows that emergency admissions associated with the consumption of ED have been increasing [6,7]. Especially the cardiovascular system seems to be affected by ED consumption: The excessive ED intake in combination with “party drugs” (i.e., illicit stimulants), alcohol, and/or in the presence of chronic medical conditions, may more frequently lead to adverse cardiovascular events such as cardiac arrhythmias or myocardial ischemia [8]. Interestingly, several clinical studies have demonstrated a significant increase in arterial blood pressure or QTc-alterations after acute ED consumption in adult subjects [9,10]. In addition, recent studies from our institution suggest that the cardiovascular system of minors may be more responsive to acute ED ingestion than that of adults [11,12]. Accordingly, the acute ED consumption led to a significantly higher blood pressure and arterial stiffness as well as a significantly lower left ventricular (LV) efficiency in juvenile study participants [12,13,14,15]. Further, we were able to demonstrate a higher disposition for cardiac arrhythmias visualized by a significantly higher prevalence of supraventricular extrasystoles after acute ED consumption [16].

Although most clinical trials have mainly focused on cardiovascular function, multiple case reports suggest that other organ systems, including the neuropsychological, hepatic, and renal system, are also affected by acute and chronic ED consumption. 

Due to the fatal outcome after excessive ED intake in an adolescent patient of our institution, we reviewed published case reports and aimed to investigate as well as summarize adverse health events in children and adolescents associated with ED consumption. Moreover, the impact of simultaneous trigger factors and/or preexisting health conditions was explored.

## 2. Materials and Methods

### 2.1. Eligibility Criteria

#### 2.1.1. Inclusion Criteria

Inclusion criteria for case reports were as follows: (1) reported patients were <18 years of age, (2) the consumption of EDs was confirmed, (3) case reports were written in the English language. No publication date restriction was defined. Any research type including relevant case report data was included.

#### 2.1.2. Exclusion Criteria

Exclusion criteria were the following: the inclusion criteria were not met; repeated publication of the same study population. 

### 2.2. Information Sources

We searched the database of PubMed, Cochrane library and Web of Science up to 9 May 2023. 

### 2.3. Search Strategy

We searched for the following text words (tw) terms, free-text terms and Medical Subject Heading (MeSH) terms individually or in combination: “Energy drink*”and “Child*” or “Adolescen*” or “Teen*” or “Youth*”and “case report*” or “Case Reports” or “Comment” or “Letter”. The search strategies for each database are available in Table 1.

### 2.4. Selection Process 

Two researchers independently screened titles and abstracts identified from the search strategy. Records, relevant articles, and reports in the references that met all inclusion criteria were fully read by the same two researchers. Full texts that did not meet the inclusion criteria were excluded. Any disagreements between the two researchers were mediated through discussions with a third researcher to reach a final decision. 

## 3. Results

### 3.1. Record Selection 

**Figure 1:**
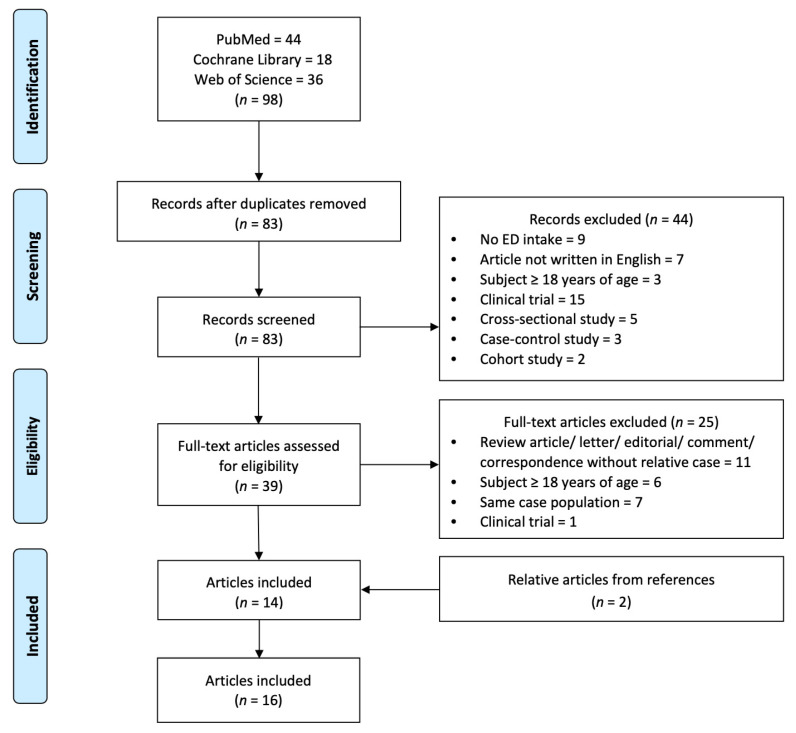
Flow diagram of literature search and selection process.

The initial search consisted of 98 records. After excluding 15 duplicated records, two researchers reviewed the titles and abstracts independently and excluded 44 records. The remaining 39 records were fully reviewed by the same two researchers and the related references were selected according to the inclusion and exclusion criteria. Ultimately, 16 records containing 17 cases were considered relevant for this review (Figure 1). 

### 3.2. Summary of Included Cases 

**Figure 2:**
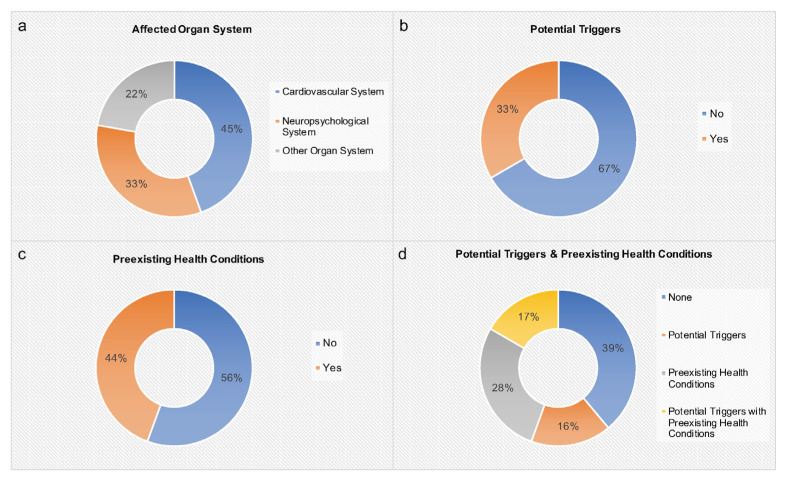
Affected organ systems, potential triggers, and preexisting health conditions associated with adverse health events after ED consumption (*n* = 18). (**a**) Affected organ system, (**b**) potential triggers (coffee/cannabis/alcohol and baseline stimulant medication/alcohol and cardiovascular exercise), (**c**) preexisting health conditions (fatigue/fainting episodes/questionable myopericarditis/history of myocarditis (confirmed by biopsy)/generalized epilepsy/ADHD, asthma, allergies/seasonal allergies/liver transplantation and re-transplantation), (**d**) potential triggers and preexisting health conditions.

A precise summary of all 16 records including 17 cases describing the age, gender, clinical presentation, and ED consumption behavior of the reported subjects is given in Table 2. In addition, our institutional case is added. Table 2 also shows information on abnormal results, diagnosis, potential triggers, preexisting health conditions, treatment, and outcome of each case. Figure 2 portrays affected organ systems, potential triggers, and preexisting health conditions associated with adverse health events after ED consumption.

## 4. Discussion

In this literature review, we determined a connection between ED intake and adverse health events in minors (Table 2). In total, 18 cases were included. Most adverse health events concerned the cardiovascular or neuropsychiatric system (Figure 2). Most cases displayed complete remission. However, based on institutional experience, one fatal outcome after excessive ED consumption was demonstrated. Interestingly, in 61% of cases potential triggers and/or preexisting health conditions were present (Figure 2).

### 4.1. Effects of Enery Drinks on the Pediatric Cardiovascular System 

Cardiovascular adverse health events were reported in 45% of cases, including cardiac arrhythmia, arterial hypertension, acute coronary artery vasospasm, and spontaneous coronary artery dissection (Table 2, Figure 2). In five subjects, potential triggers and/or preexisting health conditions were described. In the two subjects without potential triggers and/or preexisting health conditions, very large ED amounts were consumed over multiple days. One healthy male adolescent displayed spontaneous coronary artery dissection after the consumption of a reasonable ED amount (80 mg of caffeine) [17]. In this case no potential triggers were described [17]. Hence, this literature review suggests that adverse cardiovascular events associated with the consumption of EDs particularly occur if preexisting health conditions and/or additional trigger factors (e.g., alcohol, drugs, physical exercise) are present and/or very large ED amounts are consumed. 

The adverse cardiovascular health effects associated with the consumption of EDs can partially be attributed to their high caffeine content [33]. Caffeine was demonstrated to act vasoconstrictive and to increase LV inotropy leading to an augmentation of blood pressure [33]. In addition, a proarrhythmic potential of caffeinated EDs is assumed [34]. Recent studies of our department support these findings by investigating the acute effects of EDs on blood pressure, arterial stiffness, LV function, as well as heart rhythm and electrocardiographic time intervals in up to 27 healthy children and adolescents [12,13,14,15,16]. Subjects received a bodyweight-adjusted ED amount (3 mg caffeine per kg of body weight) or a placebo beverage (similar sugar content, no conventional ED ingredients) on two consecutive days [12,13,14,15,16]. Interestingly, the acute ED consumption led to significantly higher systolic and diastolic blood pressure [12,13]. Moreover, arterial stiffness of the common carotid arteries was found to be significantly higher after acute ED consumption in the examined pediatric cohort [14]. The ED consumption led to a significantly lower LV efficiency in juvenile study participants [15]. Further, a disposition for cardiac arrhythmias visualized by a significantly higher prevalence of supraventricular extrasystoles after the acute ED consumption was demonstrated [16]. In contrast to adult studies [9,35,36,37], a significant increase in the QTc interval was not observed [16]. However, it must be pointed out that in these recently published pediatric studies, the acute maximal caffeine consumption considered as safe for healthy children and teenagers by the EFSA, was not surpassed [12,13,14,15,16,38]. It remains rather unknown how the pediatric cardiovascular system reacts to higher ED amounts, particularly if additional triggers and/or preexisting health conditions are present. Moreover, due to their high sugar content the chronic intake of EDs can lead to sugar metabolism disorders and excess weight ultimately increasing long-term cardiovascular morbidity. 

In addition to caffeine, EDs may contain taurine. In the literature, the amino acid taurine is considered to decrease blood pressure and to be anti-arrhythmic [9,39,40]. The cardiovascular effects of other stimulants often added to EDs, such as B vitamins, L-carnitine or glucuronolactone, are understudied and require further research [9,33]. 

### 4.2. Effects of Energy Drinks on the Pediatric Neuropsychiatric System 

The relationship between the compounds of an ED and neuropsychiatric diseases is still rather uncharted territory. The main compound caffeine may reduce the epileptological threshold if given in low dosage and chronic use may have a protective effect [37,41]. Another example presents caffeinated analgetic medication for diverse types of headaches [42]. In contrast, it has been suggested that caffeine as a stimulant may trigger psychotic episodes and panic disorders [43]. Caffeine consumption may lead to a state of hyperexcitability in the brain potentially causing adverse reactions. A common disturbance from daily intake of caffeinated EDs may be the presence of chronic headaches due to overexcitability of the cortex [44]. A rising concern regarding EDs is their link to seizures. A study reviewing 4 adult cases of new-onset seizures found that they all consumed EDs that contained caffeine and taurine. The seizures resolved when subjects abstained from ED consumption [45].

Currently there are four caffeine-induced disorders recognized in the Diagnostic and Statistical Manual of Mental Disorders 4th edition for psychiatric health [46]. These include caffeine-induced anxiety, caffeine-related disorder, caffeine-induced sleep disorder, and caffeine intoxication [47]. A randomized placebo-controlled study, involving 90 professional athletes, indicated that insomnia, activity, and nervousness increased after ED consumption [48]. Another study examined over 1727 5th–12th grade students and concluded that children who consumed EDs had a 66% increased risk of hyperactivity and inattention compared to students who did not consume caffeine [49]. 

The amount of caffeine contained in EDs varies considerably. The pharmacokinetics of caffeine are mainly due to its antagonism of A1 and A2 adenosine receptors, preventing the neuron from blocking the release of glutamate and dopamine potentially leading to a lower seizure threshold [8]. The resulting cardiovascular effects are well studied, and it is suggested that EDs may lead to a reduction in cerebral blood flow [50], potentially triggering headaches. Finally, the literature supports the notion that ED consumption, particularly in adolescents, has adverse effects on the developing brain, possibly leading to insomnia, hyperactivity, and attention deficit disorder [51].

To the best of our knowledge, there are no controlled studies investigating the acute effects of EDs on the neuropsychological system in healthy children and teenagers so far.

### 4.3. Effects of Energy Drinks on Other Pediatric Organ Systems 

#### 4.3.1. Hepatic System

Two cases affecting the hepatic system were identified (Table 2). EDs are often highly enriched in niacin. The pediatric EFSA reference value for niacin ranges between 12 mg and 19 mg per day [52]. Five hundred mL of ED can contain around 40 mg of niacin, which is about 200% of the reference value. It is assumed that the excessive ingestion of niacin can lead to drug-induced liver injury as described in the case by Apestegui et al. (2011) [29]. However, it should be noted that in this particular case the subject received multiple liver transplantations and was therefore predisposed to liver toxins [29]. Another case described the onset of non-alcoholic steatohepatitis (NASH) after chronic ED consumption. EDs contain large amounts of sugar. In a common 16 oz ED, the sugar content can range between 37 g and 62 g, making EDs a main source of empty calories [53]. In addition, there are data implicating that caffeine intake may decrease insulin sensitivity [44]. The chronic consumption of EDs can lead to the development of sugar metabolism disorders and excess weight, which are both considered as risk factors for NASH [54]. The case of Robin et al. (2017) underlines these considerations [30]. The long-term ED intake was made responsible for the NASH onset in the obese 17-year-old subject. The initiated lifestyle modifications, including the restraint from ED consumption, led to partial remission [30]. 

#### 4.3.2. Renal System

A case reported by Schöffl et al. (2011) linked excessive ED and alcohol consumption with acute renal failure in a 17-year-old boy [31]. EDs contain large amounts of taurine. It is suggested that 95% of taurine is metabolized in the kidneys. Taurine is considered to modify renal blood flow and control osmolarity in the renal medulla [55]. The authors assume the excessive taurine intake to be potentially involved in the pathophysiology of acute renal failure by inducing tubular necrosis [31]. However, the negative effects of excessive alcohol consumption on the renal system (e.g., rhabdomyolysis) should also be considered in this case [56]. The chronic ED consumption can lead to arterial hypertension, sugar metabolism disorders and excess weight, all of which are known risk factors for chronic kidney disease [57].

#### 4.3.3. Skin

One case by Yazdi et al. (2008) described the presence of a multilocular lichen aureus in an 11-year-old boy [32]. Interestingly, the lesions resolved after the regular ED consumption was ceased by the subject. While the pathophysiology of this disease is rather unknown, the authors speculate ED ingredients to be a potential trigger of its onset [32]. 

### 4.4. Prevention of Adverse Health Events Associated with Energy Drink Consumption 

While children and teenagers represent a large percentage of the ED consumer market, studies examining the effects of their consumption; acute or chronic, on pediatric health are lacking. Medical associations, such as the American Academy of Pediatrics apprehend a potential health risk in association with these beverages in minors and advise against ED consumption [58]. Furthermore, several countries, including Lithuania and Latvia, banned the sale of EDs to minors [59,60]. To reduce future adverse reactions connected to ED intake, children and adolescents should be informed about the associated risk factors and responsible ED consumption behaviors. Minors with preexisting health conditions should be discouraged from drinking EDs. In addition, ED consumption in combination with potential trigger factors (e.g., physical activity, long-term medication, drug/alcohol abuse) should be avoided. 

### 4.5. Limitations 

A potential limitation of this review is publication bias. With 18 cases included, this literature review can be considered relatively small. In addition, in some cases, the exact ED ingredients, their concentrations, or the specific ED amount were not described. In addition, in some cases, information about specific trigger factors (e.g., physical activity, illicit drugs, etc.,) was not reported. It is possible that some subjects withheld information about concomitant drug use. These limitations must be taken into consideration, as they could have altered the demonstrated results.

## 5. Conclusions

This literature review suggests that ED consumption may well be associated with adverse health events in minors. The cardiovascular and the neuropsychiatric systems seem to be predisposed. Particularly, ED consumption in combination with potential trigger factors or in the presence of preexisting health conditions appears to be critical. To prevent adverse health effects in the future, children and adolescents should be informed about ED-associated risk factors and responsible ED consumption behaviors. Minors with preexisting health conditions should be discouraged from drinking EDs. In addition, ED consumption in combination with potential trigger factors (e.g., physical activity, drug intake) should be avoided. To better understand and determine the possible side-effects of ED consumption in minors with or without preexisting health conditions and to investigate possible trigger factors, we highlight the necessity of future controlled studies and clinical trials. The validated data may not only aid the acute clinical manifestations but also support the primary prevention.

## Figures and Tables

**Table 1:** Search strategies.

Search Number	Queries
Queries in PubMed	
#1	Search (Energy drink * [tw])
#2	Search (Child * [tw])]) OR (Adolescen * [tw]) OR (Teen * [tw]) OR (Youth * [tw])
#3	Search (case report * [tw]) OR (Case Reports [Publication Type]) OR (Comment [Publication Type]) OR (Letter [Publication Type])
#4	#1 AND #2 AND #3
Queries in Cochrane	
#1	(“energy drink *”): ti, ab, kw
#2	(“child *”): ti, ab, kw OR (“adolescen *”): ti, ab, kw OR (“teen *”): ti, ab, kw OR (“youth *”): ti, ab, kw
#3	(“case report *”): ti, ab, kw OR (comment): pt OR MeSH descriptor: [Editorial] explode all trees OR (“editorial): pt OR MeSH descriptor: [Letter] explode all trees OR (“letter”): pt
#4	#1 AND #2 AND #3
Queries in Web of Science	
#1	TS = (Energy drink *)
#2	TS = (child *) OR TS = (adolescen *) OR TS = (teen *) OR TS = (youth *)
#3	TS = (case report *)
#4	#1 AND #2 AND #3

* Wildcard for capture variations of search terms. Tw, text word; ti, title; ab, abstract; kw, keyword; MeSH, Medical Subject Headings; pt, publication type; TS, topic.

**Table 2:** Summary of included cases associated with adverse health events in children and adolescents after the consumption of energy drinks.

Author, Year	Age, Sex	Clinical Presentation	ED Consumption Behavior	Abnormal Results	Diagnosis	Potential Triggers	Preexisting Health Conditions	Treatment	Outcome
Cardiovascular system									
Polat et al., 2013 [17]	13, M	Acute mid-sternal chest pain	An ED (80 mg caffeine) for the first time the night before admission	Cardiac auscultation: S4 gallop; ECG: 2–3 mm ST-segment elevations in leads II, III, aVF and V3 through V5; dynamic T-wave changes in leads V3–V5 24 h after the onset; TEE: LVEF about 54% with moderate apical hypokinesis; coronary angiography: extensive dissection with a visible tear from the distal part of the LAD artery	SCAD with STEMI	None	None	Acetylsalicylic acid, subcutaneous enoxaparin, sublingual nitroglycerin, enalapril, metoprolol	Complete remission
Dufendach et al., 2012 [18]	13, F	Palpitations, chest pain, shakiness, and dizziness	At least 1 16 oz ED (160 mg caffeine) every other day for 2 weeks prior to presentation	ECG: idiopathic extreme QT-prolongation; genetic testing: LQT1-causative mutation (G179S-*KCNQ1*)	Type 1 LQTS	None	Two “possible” fainting episodes	Beta-blockers	Partial remission
Di Rocco et al., 2011 [19]	14, M	Persistent “heart fluttering”	Unknown ED amount the day before presentation	Irregular HR of 130/min with a 1/6 vibratory systolic ejection murmur; ECG: narrow-complex tachycardia with atrial fibrillation and occasional atrial flutter	Arrhythmia	Running race	None	Digoxin	Complete remission
Di Rocco et al., 2011 [19]	16, M	Intoxication, vomiting after minor head trauma	Unknown ED amount mixed with vodka the day prior to admission	Irregular HR of 160/min; ECG: chaotic atrial tachycardia and atrial fibrillation with rapid ventricular response; blood ethanol level: 155 mg/dL	Arrhythmia	Alcohol, baseline stimulant medication (amphetamine, dextroamphetamine, montelukast, loratadine, doxycycline)	ADHD, asthma, allergies	A bolus of normal saline and intravenous fluid support	Complete remission
Usman et al., 2012 [20]	16, M	Palpitations for one week and elevated BP	80–100 ED cans in two weeks, at least 3 ED cans the day prior to presentation	BP: 150/95 mmHg; regular pulse of 110/min	Arterial hypertension; sinus tachycardia	None	None	None	Complete remission
Terlizzi et al., 2008 [21]	16, F	Three months of orthostatic intolerance and episodes of transient loss of consciousness	4–5 ED cans per day one week before symptom manifestation	A sharp increase in HR during head up tilt test from 88 to 128 bpm, at the 22nd minute BP and HR decreased and the patient referred dizziness, blurred vision, and malaise	Postural tachycardia syndrome	None	None	None	Complete remission
Own institutional experience	16, F	Collapse, unconsciousness	Multiple ED cans days prior to event	Refractory ventricular fibrillation	Refractory ventricular fibrillation, circulatory failure, consecutive hypoxic brain damage	Lack of sleep, anxiety	History of myocarditis (confirmed by biopsy)	CPR, ECMO	Fatal outcome
Wilson et al., 2012 [22]	17, M	Acute chest pain with radiation to the left arm	5–7 ED cans (560–800 mg caffeine) the night prior to admission	ECG: Diffuse ST-segment elevations in leads II, III, AVF, V3–V6, and ST-segment depressions in leads V1 and AVR; elevated WBC; reduced potassium and bicarbonate; elevated CK and troponin T; TEE: LVEF at 50% with apical hypokinesis	Acute coronary artery vasospasm	None	Questionable myopericarditis	Acetylsalicylic acid, nitroglycerin, diltiazem	Complete remission
Neuropsychological system									
Butragueño Laiseca et al., 2019 [23]	8, M	Paraesthesia around the oral commissure, clonus and commissure deviation, difficulty speaking and retaining saliva	1 ED on daily basis, increased intake the week prior to admission	Electroencephalography: bihemispheric epileptiform activity, predominantly during non-REM sleep	Rolandic epilepsy	None	None	Oxcarbazepine	No remission
Staikoglou et al., 2022 [24]	14, M	Dysarthria, headache, mild right-hand weakness, hypesthesia and right optic field deficits	2 L of ED within 10 h prior to admission	BP: 190/120 mmHg, HR: 116/min, MRI: a typical “string and pearls” sign of the left P2 and P3 posterior cerebral artery segment	Brain ischemia secondary to dissection of posterior cerebral artery	None	None	Anticoagulation therapy	Partial remission
Babu et al., 2011 [25]	15, M	Tonic-clonic seizure, postictal state, repeated vomiting	2 ED bottles and a cup of coffee within 2 h	Temperature: 38.1 °C; pulse: 120/min, respiratory rate: 40/min; reduced potassium; serum caffeine level: 99 µg/mL; brain MRI: mild ethmoid sinus disease	Tonic-clonic seizure	Additional consumption of a cup of coffee	Seasonal allergies	Intravenous lorazepam, intravenous ondansetron and normal saline	Complete remission
Yamada-Takeda et al., 2019 [26]	16, M	Breakthrough seizure	9 g ED powder 2 times per day	A significant drop of valproic acid; probable drug-herb interaction (score of drug interaction probability scale: 5)	Breakthrough seizure	None	Generalized epilepsy and seizure-free for 2 years with divalproex extended-release	None	Complete remission
Samanta D, 2015 [27]	16, M	Thunderclap headache, vomiting, left leg numbness and gait difficulty	4 × 8 oz ED can (320 mg caffeine) several hours before the onset of symptoms	Tachycardia, brisk deep tendon reflexes of the left knee and ankle, positive Babinski sign of the left side; brain MRI: numerous cortical and subcortical foci of abnormally restricted diffusion; MR angiography: diffuse luminal irregularity with intermittent narrowing of distal branches of posterior cerebral artery	Reversible cerebral vasoconstriction syndrome	None	None	Oral analgetic and antiemetic medication, verapamil	Complete remission
Quadri et al., 2018 [28]	17, F	New onset mania for 5 days	1 or 2 16 oz ED containers (300–600 mg caffeine) per day over 7 days prior to admission	Urine drug screen: positive for cannabis; quantitative THC: 198 ng/mL	Caffeine-induced bipolar disorder	Prior cannabis use	None	Olanzapine	Partial remission
Other organ systems									
Apestegui et al., 2011 [29]	16, M	Jaundiced	15 cans of ED within 3 days 2 weeks before the first hepatitis episode; 3 cans of ED within 4 h 2 days prior to the second hepatitis episode	Elevated AST, ALT, BIL and GGT; biopsy sample: severe perivenular hepatocellular necrosis, major centrilobular and portal inflammation, minor signs of endotheliitis and cholangitis	Cholestatic hepatitis	None	Liver transplantation due to biliary tumor, retransplantation due to biliary tract lesions	Unchanged low-dose tacrolimus monotherapy	Partial remission
Robin et al., 2017 [30]	17, M	Elevated ALT	Chronic consumption of 6 × 500 mL ED cans per day	Elevated ALT up to 274 U/L; ultrasound: moderate liver steatosis; liver biopsy: moderate steatosis and mild steatohepatitis	NASH	None	Fatigue	Change of diet and lifestyle	Partial remission
Schöffl et al., 2011 [31]	17, M	Vomiting, dizziness, hyperventilating and capsular pain over both kidneys	3 L ED (780 mg caffeine and 4600 mg taurine) mixed with 1 L vodka (380 g alcohol) over the course of an evening	Tachycardiac with an HR of 110/min, transient hypertension, serum creatinine: 6.9 mg/dL, urine sediment revealed acute tubular necrosis	Acute renal failure	Alcohol, 2 × 100 m running races	None	Hemodialysis, enalapril	Complete remission
Yazdi et al., 2008 [32]	11, M	Asymptomatic lesions at both upper and lower extremities for 5 months	1 ED daily	Physical examination: multiple ochre macules and patches with central petechiae localized on limbs and the lower abdomen; biopsy: epidermal acanthosis with intra-epidermal collection of lymphocytes; iron-stain detection of hemosiderin: lymphocytic infiltrate of the upper dermis containing hemosiderin deposits and extravasal erythrocytes adjacent to dermal blood vessels; PCR of T-cell receptor-γ: clonal rearrangement	Lichen aureus	None	None	ED abstinence	Complete remission

ADHD: attention deficit hyperactivity disorder, ALT: alanine aminotransferase, AST: aspartate aminotransferase, BIL: bilirubin, BP: blood pressure, CK: creatine kinase, CPR: cardiopulmonary resuscitation, ECG: electrocardiogram, ECMO: extracorporeal membrane oxygenation, ED: energy drink, F: female, GGT: gamma-glutamyltransferase, HR: heart rate, LAD: left anterior descending artery, LQTS: Long QT syndrome, LQT1: Long QT syndrome type 1, LVEF: left ventricular ejection fraction, M: male, MRI: magnetic resonance imaging, NASH: non-alcoholic steatohepatitis, PCR: polymerase chain reaction, REM: rapid eye movement, SCAD: spontaneous coronary artery dissection, STEMI: ST-segment elevation myocardial infarction, THC: tetrahydrocannabinol, TTE: transthoracic echocardiography, WBC: white blood count.

## Data Availability

No datasets were generated or analyzed during the current study.
